# Association Between COVID-19 Pregnant Women Symptoms Severity and Placental Morphologic Features

**DOI:** 10.3389/fimmu.2021.685919

**Published:** 2021-05-26

**Authors:** Patricia Zadorosnei Rebutini, Aline Cristina Zanchettin, Emanuele Therezinha Schueda Stonoga, Daniele Margarita Marani Prá, André Luiz Parmegiani de Oliveira, Felipe da Silva Dezidério, Aline Simoneti Fonseca, Júlio César Honório Dagostini, Elisa Carolina Hlatchuk, Isabella Naomi Furuie, Jessica da Silva Longo, Bárbara Maria Cavalli, Carolina Lumi Tanaka Dino, Viviane Maria de Carvalho Hessel Dias, Ana Paula Percicote, Meri Bordignon Nogueira, Sonia Mara Raboni, Newton Sergio de Carvalho, Cleber Machado-Souza, Lucia de Noronha

**Affiliations:** ^1^ Postgraduate Program of Health Sciences, School of Medicine, Pontifícia Universidade Católica do Paraná-PUCPR, Curitiba, Brazil; ^2^ Postgraduate Program in Biotechnology Applied in Health of Children and Adolescent, Pelé Pequeno Príncipe, Research Institute, Faculdades Pequeno Príncipe, Curitiba, Brazil; ^3^ Department of Medical Pathology, Clinical Hospital, Universidade Federal do Paraná-UFPR, Curitiba, Brazil; ^4^ Department of Tocogynecology, Clinical Hospital, Universidade Federal do Paraná, UFPR, Curitiba, Brazil; ^5^ Postgraduate Program of Tocogynecology and Women’s Health, Clinical Hospital, Universidade Federal do Paraná-UFPR, Curitiba, Brazil; ^6^ Virology Laboratory, Clinical Hospital, Universidade Federal do Paraná-UFPR, Curitiba, Brazil; ^7^ Department of Infectious Disease, Clinical Hospital, Universidade Federal do Paraná-UFPR, Curitiba, Brazil

**Keywords:** SARS-CoV-2, COVID-19, vertical transmission, placenta, morphometric analysis, placental histopathology

## Abstract

Since the beginning of the pandemic, few papers describe the placenta’s morphological and morphometrical features in SARS-CoV-2–positive pregnant women. Alterations, such as low placental weight, accelerated villous maturation, decidual vasculopathy, infarcts, thrombosis of fetal placental vessels, and chronic histiocytic intervillositis (CHI), have been described.

**Objective:**

To analyze clinical data and the placental morphological and morphometric changes of pregnant women infected with SARS-CoV-2 (COVID-19 group) in comparison with the placentas of non-infected pregnant women, matched for maternal age and comorbidities, besides gestational age of delivery (Control group).

**Method:**

The patients in the COVID-19 and the Control group were matched for maternal age, gestational age, and comorbidities. The morphological analysis of placentas was performed using Amsterdam Placental Workshop Group Consensus Statement. The quantitative morphometric evaluation included perimeter diameter and number of tertiary villi, number of sprouts and knots, evaluation of deposition of villous fibrin, and deposition of intra-villous collagen I and III by Sirius Red. Additionally, Hofbauer cells (HC) were counted within villi by immunohistochemistry with CD68 marker.

**Results:**

Compared to controls, symptomatic women in the COVID-19 group were more likely to have at least one comorbidity, to evolve to preterm labor and infant death, and to have positive SARS-CoV-2 RNA testing in their concepts. Compared to controls, placentas in the COVID-19 group were more likely to show features of maternal and fetal vascular malperfusion. In the COVID-19 group, placentas of symptomatic women were more likely to show CHI. No significant results were found after morphometric analysis.

**Conclusion:**

Pregnant women with symptomatic SARS-CoV-2 infection, particularly with the severe course, are more likely to exhibit an adverse fetal outcome, with slightly more frequent histopathologic findings of maternal and fetal vascular malperfusion, and CHI. The morphometric changes found in the placentas of the COVID-19 group do not seem to be different from those observed in the Control group, as far as maternal age, gestational age, and comorbidities are paired. Only the deposition of villous fibrin could be more accentuated in the COVID-19 group (p = 0.08 borderline). The number of HC/villous evaluated with CD68 immunohistochemistry did not show a difference between both groups.

## Introduction

One year after the recognition of the outbreak of the severe acute respiratory distress syndrome coronavirus 2 (SARS-CoV-2), it has spread all over the world, thus developing into a global pandemic with nearly 150 million confirmed infections and more than 3.1 million deaths worldwide so far (1). Fatalities and severe courses were primarily seen in elderly patients with relevant comorbidities, but soon there were reports of younger patients showing adverse outcomes (2, 3).

No longer after its first documented appearance, SARS-CoV-2 was suspected to be perinatally transmitted (4–8). This enveloped single-stranded RNA virus infects target cells by binding to angiotensin-converting enzyme 2 (ACE2) and entry into cells after spike protein cleavage by the transmembrane serine protease 2 (TMPRSS2). Since both proteins have been detected in the placenta and fetal tissues, a possible mechanism of intrauterine transmission and neonatal infection emerged (9–14), and the coronavirus disease 2019 (COVID-19) impact on pregnant women became of particular interest.

Along with the worldwide dissemination of COVID-19, reports of adverse pregnancy outcomes have emerged in the literature, such as preeclampsia, preterm delivery, miscarriage, intrauterine fetal demise, and neonatal death (15–18). Congenital infection can be challenging to characterize since pathogen detection usually requires specific methods, not always available, applied in a myriad of maternal and fetal samples. Despite that, antepartum or peripartum vertical transmission is now substantially documented. Almost 30% of neonatal infections reported to date occurred due to transplacental transmission, and the remaining due to environmental exposure. Additionally, Raschetti et al. observed that 55% of infected neonates developed COVID-19 (19–25).

It is well recognized that analysis of the placental histopathological changes can provide valuable information, considering that a variety of pathological agents, including infectious ones, are associated with characteristic morphological findings (26–29). Regardless, few papers describe the placenta’s morphological and morphometrical features in SARS-CoV-2–positive pregnant women (30–34), and the association between maternal infection and abnormal placental findings is still to be determined.

Accordingly, the purpose of this study was to analyze clinical data and the morphological and morphometric changes in placentas of pregnant women infected with SARS-CoV-2 (COVID-19 group) and to compare the placentas of non-infected pregnant women (Control group) matched in a 1:1 fashion by gestational age at delivery, maternal age, and comorbidities.

## Materials and Methods

### Ethical Approvals

The Brazilian National Ethics Committee approved the presented study of Human Experimentation under the protocol number CAAE: 35129820.6.0000.0096. Families signed the informed consent forms. The authors followed all relevant guidelines, regulations, and ethics and safety protocols during this study execution at all stages. The data that support the findings of this study are available from the corresponding author.

### Study Design

A prospective observational case-control study.

### Study Patients and Control Group Selection

For the COVID-19 group (study group), pregnant women with laboratory-confirmed infection, whose respective placenta specimens have been sent for histologic examination, were eligible for inclusion. This group comprises 19 women who had SARS-CoV-2 infection confirmed either in the second (n = 3) or in the third gestational trimester (n = 16). Initially, we included women who spontaneously sought treatment at Complexo Hospital de Clínicas, Universidade Federal do Paraná (CHC-UFPR) or at Hospital Nossa Senhora das Graças (HNSG), Curitiba, Brazil, for symptoms of COVID-19 varying from mild to severe (n = 9). After the implementation of universal testing for SARS-CoV-2 infection for all obstetrical patients admitted to labor and delivery in both institutions, asymptomatic pregnant women were added thereafter (n=10).

For the Control group (n = 19), placentas of pregnant women who gave birth at CHC-UFPR in years prior to the SARS-CoV-2 outbreak were selected (from 2016 to 2018) and matched in a 1:1 fashion by gestational age at delivery, maternal age, and maternal comorbidities.

Historical controls were selected to ensure that women with false-negative test results for SARS-CoV-2 infection were excluded. Gestational age at delivery is a universally used matching variable since there is a correlation between placental development and the advancing gestation, which could interfere in the analysis. Maternal age and maternal comorbidities were also incorporated as matching variables, given their possible role as confounders when analyzing placental abnormalities.

### Samples

Submission criteria for placental examination included maternal or fetal conditions previously diagnosed during prenatal care or gross abnormalities noted during delivery, as routinely implemented in both institutions. Therefore, the maternal-positive SARS-CoV-2 testing result was considered an abnormal maternal condition and a placental evaluation criterion.

All placentas were examined according to a standardized protocol that consisted of immediate fixation after delivery in 10 % buffered formalin for 72 h, gross examination with measurement of placental dimensions and chord length, weight evaluation of the placental disc after trimming of the fetal membranes and umbilical cord, followed by serial sectioning through 1.5-cm interval and cut surface examination. Macroscopic alterations were recorded and sampled. Additional representative samples of the umbilical cord (two sections), the membranes (one fetal membrane roll), and the chorionic plate (at least two full-thickness, non-peripheral sections including maternal and fetal surface) were submitted to paraffin embedding.

### Clinical Information

Clinical and laboratory data were obtained from medical records during hospitalization; the mother and the newborn were followed up until discharge from the hospital.

Maternal information sought from each medical record for both groups comprised maternal age and comorbidities, parity, gestational age at delivery, mode of delivery, neonatal birth weight, and APGAR score. All pregnant women in both groups were tested for congenital intrauterine infections (TORCH) during prenatal care; only one (Patient code—PC 20-3744) tested positive for syphilis in the first trimester and received the preconized treatment.

For the study group, it was also retrieved the gestational age at and method used for SARS-CoV-2 infection diagnosis, presence or absence of symptoms typically attributed to COVID-19 (body temperature over 38°C, dyspnea, cough, myalgias, nausea and vomiting, diarrhea, headache, anosmia), disease severity (ranging from mild symptoms to critical organ dysfunction), and maternal outcome.

### SARS-CoV-2 Testing

Maternal nasopharyngeal swabs specimens (n = 17) were tested for SARS-CoV-2 infection by real-time reverse transcriptase-polymerase chain reaction (RT-PCR). In the remaining two cases, the diagnosis was achieved by serologic testing (n = 2).

Detection of SARS-CoV-2 RNA in infants was performed in 13 case samples (n = 13), including umbilical cord blood (n = 13), amniotic fluid (n = 3), and infants’ nasopharyngeal swabs specimens (n = 6), all of them collected immediately after birth. The mothers’ milk was also tested in three cases.

Samples from formalin-fixed paraffin-embedded (FFPE) tissue were also tested to verify the presence of SARS-CoV-2 RNA in the placenta (n=11). Viral RNA extraction was performed with a commercially available paraffin extraction kit (Qiagen^®^) in Pelé Pequeno Príncipe Research Institute.

SARS-CoV-2 RNA identification tests were performed at CHC-UFPR and HNSG using XGEN MASTER COVID-19 Kit (Mobius Life Science, Inc, Brazil). Quantitative anti-SARS-CoV-2 IgM and IgG dosage was done in peripheral blood samples in both institutions.

### Morphologic Analysis

All representative placental samples taken for microscopic assessment underwent routine processing, embedding, sectioning at 5 µm, and staining with hematoxylin and eosin (H&E).

The qualitative morphological analysis was performed in all placentas from the COVID-19 and Control groups (n = 38) using the Amsterdam Placental Workshop Group Consensus Statement (35). Histological sections containing at least one sample of the umbilical cord, membrane roll, and chorionic plate of every case from both groups were randomly selected and renamed by one research team member (blinding step). Two experienced perinatal pathologists systematically evaluated the slides.

Selected parameters were computed simply as “present” (yes) or “absent” (no). The extension and intensity of specific alterations were graded following Amsterdam protocol recommendations. The remaining parameters were subdivided into three quantitative categories: <30%, 30% to 70%, and > 70%, to record both the presence and extension of most alterations.

Assessed parameters included features of maternal vascular malperfusion (villous infarction, distal villous hypoplasia, accelerated villous maturation/increase in syncytial knots, decidual acute atherosis, decidual vascular fibrinoid necrosis with or without foam cells, decidual vascular mural hypertrophy, decidual chronic perivasculitis, absence of spiral artery remodeling, decidual arterial thrombosis, persistence of intramural endovascular trophoblast), features of fetal vascular malperfusion (fetal vascular thrombosis, fetal vascular intramural fibrin deposition, avascular villi, stem vessel obliteration/fibromuscular sclerosis, villous stromal-vascular karyorrhexis, vascular ectasia), delayed villous maturation, features of maternal inflammatory response (chorionitis, chorioamnionitis), features of fetal inflammatory response (umbilical vasculitis, funisitis), features of chronic inflammation (chronic deciduous, villitis, intervillositis), intervillous thrombi, microcalcification foci, chorangiosis, and membrane meconium and hemosiderin staining.

### Morphometric Analysis

The morphometric analysis included measurement of the perimeter and diameter of the villi, counting the number of tertiary villi, sprouts and knots of tertiary villi, quantifying the villous fibrin and the intra-villous collagen I and III depositions. It was performed in 11 cases of the COVID-19 group and eleven Control group cases (n = 22), matched for gestational age, maternal age, and maternal comorbidities.

One histological section stained with H&E containing at least one sample of the chorionic plate was randomly selected from every case in both groups (n = 22). Samples of grossly identified lesions were previously excluded. Each section was subsequently photographed at a magnification of 200× (medium power field—MPF) using the Scanner Axion Scan.Z1 (Zeiss AG, Oberkochen, Germany), resulting in about 5,000 high-resolution images for each case (ZEN Blue Edition, Zeiss, Germany). Following the exclusion of unfocused images, with artifacts, with non-villous tissue, or less than 100% of the field occupied with placental villi, the remaining images were randomized to obtain about 100 images for each case of both groups. The villi’s perimeter and diameter were measured using Image-Pro Plus^®^ 4 software, based on freehand drawing on 100 villi in consecutive images. At the end of each villus’ contour, the program provided perimeter and minor diameter in micrometers (µm).

The variables number of tertiary villi, number of sprouts, and knots of tertiary villi were assessed by simply counting these microscopic structures by an experienced perinatal pathologist, in 30 of those 100 images, after a new cycle of randomization.

Histological sections were also stained with phosphotungstic hematoxylin (n = 22) and Sirius Red (n = 22), aiming to evaluate villous fibrin and intra-villous collagen I and III depositions, respectively. As previously described for H&E, the slides were photographed at MPF using the Scanner Axion Scan.Z1, and the resulting images were randomized to obtain about 200 images for every case of the COVID-19 and Control group for each stain. Positive control was chosen as a “mask,” which contained adequate levels of specific pigment precipitation. The mask was then superimposed on the sample images, and Image-Pro Plus 4 software identified the positive areas, expressing the results as positive pigment deposition areas per μm^2^. The values obtained for the collagen analysis were further divided by the observed field’s total area, generating a percentage value for each image (36).

The morphometric analysis was also blind since each case was previously renamed by one member of the research team not involved in the data acquisition, and the images were also randomly generated by the software afterward, with no investigator’s interference.

### Immunohistochemistry

Histological sections of the placentas from both groups (n = 22) were fixed on electrically charged glass slides and subsequently dewaxed with heated xylol (37°C), dehydrated by successive baths of absolute ethyl alcohol with decreasing solution concentrations and rehydrated with water. Methyl alcohol and hydrogen peroxide were used to block endogenous peroxidase and distilled water and hydrogen peroxide for the second block. Next, incubation with anti-CD68 primary antibody (KP1 clone, monoclonal mouse, Biocare, California, USA) for 1 h and secondary antibody associated with the dextran polymer (Spring Bioscience, Pleasanton, USA) for 30 min. For development, DAB/substrate complex (DAB, DakoCytomation) was added onto the slides, followed by counterstaining with Mayer’s hematoxylin, dehydration with ethyl alcohol baths in increasing concentrations, clarification with xylol, and blending with Canada balsam. The protocol developed and described above is already standardized and routinely used in CHC-UFPR. To quantify Hofbauer cells (HC), the number of villi and CD68+ cells in those villi were counted in 30 high-power random fields (HPF= 400×). Only positive cells morphologically compatible with histocytes, with visible nuclei, and located within villi were considered suitable for counting. Unspecific staining, staining of any other cells, and cells without visible nuclei were excluded.

All immunohistochemistry assays included a negative control (missing a primary antibody) and positive control (human lymph node).

### Statistical Analysis

Means, standard deviations, medians, minimum, maximum values, frequencies, or percentages were used to describe the findings. The nominal variables are expressed as actual values and frequency and analyzed by Pearson chi-square test and or by Fisher exact test. Most of the quantitative variables exhibited normal distribution, as verified by the Shapiro–Wilk test, and were compared with the t-test. The Kruskal–Wallis non-parametric test was performed to compare the remaining quantitative variable between groups (fibrin deposition). For both tests, statistical significance was defined as a *p*-value of <0.05. The data were analyzed using the IBM SPSS Statistics v.20.0 software. Armonk, NY: IBM Corp.

## Results

**Figure 1:**
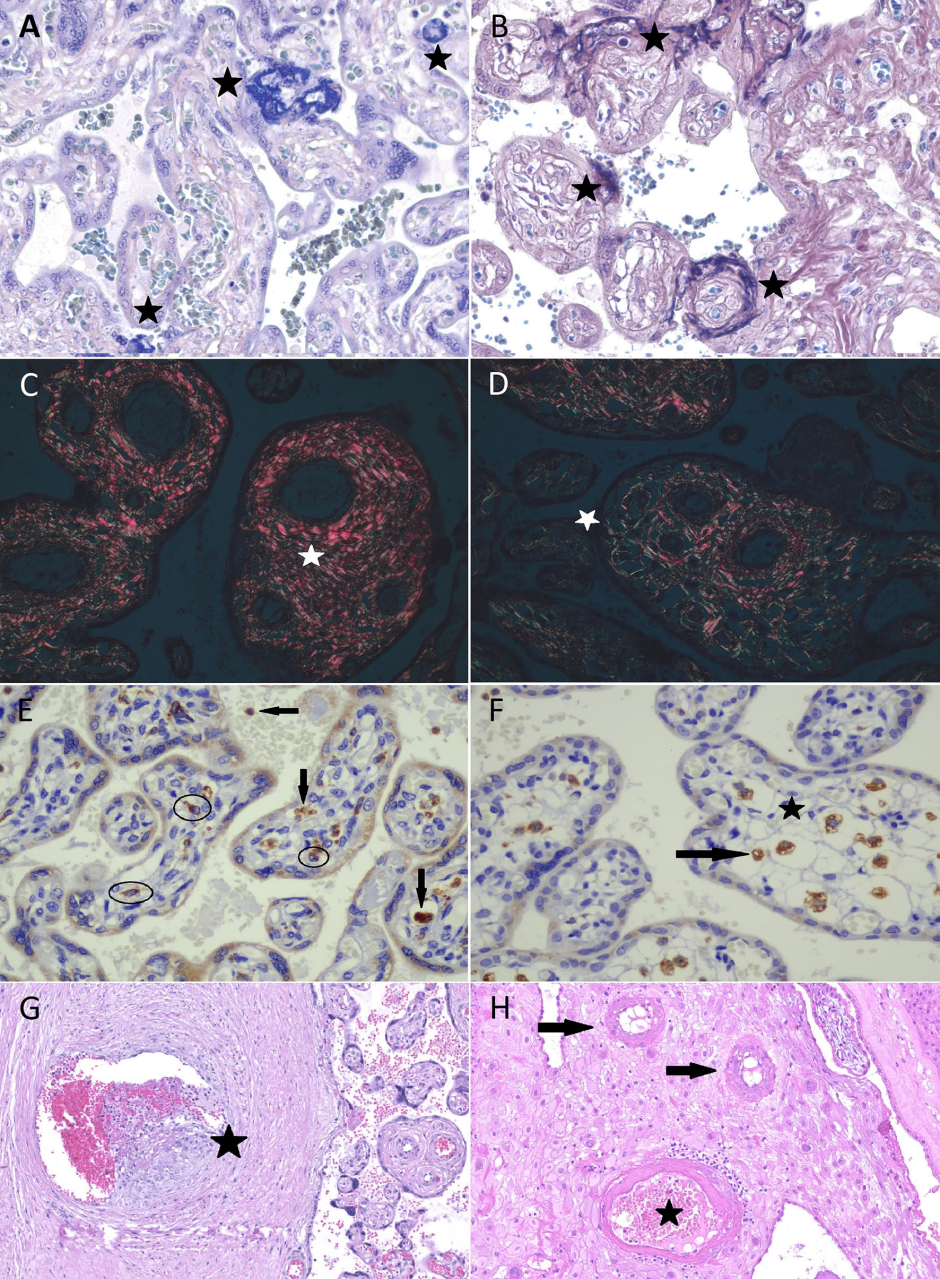
Morphometric and morphological analysis of placental specimens from women infected with SARS-CoV-2 (COVID-19 group) and the Control Group. Fibrin deposition evaluation in COVID-19 group **(A)** and Control group **(B)** in phosphotungstic hematoxylin (star—deep blue amorphous material); both perivillous and intravillous deposition were included. Sirius Red: bright red collagen I **(C)** and green collagen III fibers **(D)** under polarized light. Photomicrography of immunostaining with CD68 (KP1 Clone, Biocare) in COVID-19 group **(E)** and in Control group **(F)**; eligible Hofbauer cells to counting (circled cells and those near the star). Macrophages outside villi and unspecific marking were excluded (arrows). Fetal vascular thrombosis **(G)** and decidual vasculopathy **(H)** in COVID-19 cases.

Relevant clinical information about each case in both groups is summarized in **Tables 1** and **2**. Comparison between groups regarding clinical data and morphologic findings are resumed in **Table 3** (n = 38). In **Table 4**, morphometric data are presented along with clinical information of the cases evaluated from both groups (n = 22). **Figure 1** exemplifies morphometric and morphological parameters evaluated.

**Table 1:** Clinical information of the COVID-19 group.

Patient code	Maternal age (yr)	Maternal Comorbidities	COVID-19 Symptoms/Severe Disease	SARS-CoV-2 testing			Outcome			
				RT-PCR Maternal NS swab/Trimester	Maternal Serology	RT-PCR placenta/RT-PCR fetal samples	Gestational age at delivery	APGAR (1 min/5 min)/Fetal-Maternal Outcome	Fetal weight (g)	Placental weight (g)/Macroscopic alterations
**20–3594**	26	Hypertensive disorder in pregnancy and hypothyroidism	+/+	+/3rd	IgM+/IgG+	−/−	33	(5/9) Preterm newborn	2450	448/Infarcts (<5%)
**20–3561**	38	Hypothyroidism	+/+	*/2nd	IgM+/IgG+	−/−	28+2	(na) Preterm newborn	na	245
**20–3282**	40	*Situs inversus totalis* with metallic stent	+/+	+/3rd	na	−/+**	33+5	(0/0) Neonatal death/Maternal death	2300	416
**20–5379**	38	Gestational diabetes	+/+	+/2nd	na	−/+**	23+6	(1/5) Neonatal death/Maternal death	610	168/Placental hypoplasia
**20–3744**	29	Gestational diabetes, hypothyroidism, obesity, bipolar disorder, and syphilis (treated)	+/−	+/3rd	IgM+/IgG+	−/+**	34+1	(na) Preterm newborn	na	412/Infarcts (<5%)
**20–5105**	29	None	−	+/3rd	na	−/na	38+6	(9/10) Term newborn	2960	462
**20–3369**	29	Gestational diabetes and Hyperthyroidism	+/−	+/3rd	na	−/na	37+4	(8/9) Term newborn	2600	358/Infarcts (<5%)
**20–3364**	42	Hypertensive disorder in pregnancy	+/+	+/2nd	IgM+/IgG+	+/+^§^	28+3	Intrauterine death	1020	135/Placental hypoplasia and infarcts (30–40%)
**20–5776**	42	None	−	+/3rd	na	−/+**	36+5	(9/10) Preterm newborn	2605	382
**20–5869**	27	None	+/−	+/3rd	na	−/na	37+2	(9/10) Term newborn	2345	370
**20–3916**	24	None	−	+/3rd	IgM+/IgG+	−/na	38+6	(9/10) Term newborn	3030	650
**20–4850**	25	Obesity	+/+	+/3rd	na	na/−	30+2	(na) Preterm newborn	na	410
**20–5006**	22	Obesity	−	+/3rd	na	na/−	41+0	(4/9) Term newborn	3110	670
**20–5009**	23	Hypothyroidism	−	*/3rd	IgM+/IgG+	na/−	38+4	(5/9) Term newborn	3925	775/Hydropic placenta
**20–5031**	35	Gestational diabetes, obesity	−	+/3rd	na	na/−	36+4	(8/9) Preterm newborn	2720	450
**20–6551**	32	None	−	+/3rd	na	na/na	38+5	(8/9) Term newborn	3070	448
**20–6680**	38	None	−	+/3rd	na	na/na	37	(9/10) Term newborn	2875	318
**20–7035**	34	None	−	+/3rd	na	na/−	39	(9/9) Term newborn	3115	438
**20–6071**	34	Hypertensive disorder in pregnancy	−	+/3rd	na	na/−	34+6	(7/8) Preterm newborn	2370	384

*rt-PCR not available—diagnostic by serology.

RT-PCR positive in fetal samples: **Nasofaringeal swab and ^§^Umbilical cord blood.

na, not available.

**Table 2:** Clinical information of the Control group.

Patient code	Maternal age (yr)	Maternal Comorbidities	Outcome			
			Gestational age at delivery	APGAR (1 min/5 min)/Fetal Outcome	Fetal weight (g)	Placental weight (g)Macroscopic alterations
**16–7859**	20	Hypothyroidism	32+3	(3/7) Preterm newborn	1180	270/Placental hypoplasia
**18–13016**	23	Chronic hypertension and hypothyroidism	35+2	(4/8) Preterm newborn	2223	498/none
**16–8315**	18	Obesity	40+4	(7/9) Term newborn	3810	514 none
**18–4906**	20	None	28	(7/8) Preterm newborn	1205	248/none
**18–14057**	42	Diabetes, chronic hypertension, bipolar disorder	33+4	(2/8) Preterm newborn	1650	243/Placental hypoplasia
**16–7599**	25	Gestational diabetes	39	(8/10) Term newborn	3460	480/none
**16–3340**	39	None	38+3	(7/9) Term newborn	3005	395/none
**18–9951**	24	None	37+2	(8/9) Term newborn	3690	574/none
**16–6144**	29	None	39	(9/10) Term newborn	3345	394/none
**17–2491**	35	None	32+4	(8/9) Preterm newborn	2900	416/none
**16–7667**	36	None	36+4	(9/9) Preterm newborn	2315	319/none
**18–5040**	24	None	32+4	(1/6/8) Preterm newborn	1555	297/Infarcts (5%)
**16–7155**	25	Obesity	40	(7/9) Term newborn	2830	375/none
**16–5762**	39	Hypothyroidism	39+3	(8/9) Term newborn	3490	465/Infarcts (10%)
**18–11859**	38	Gestational diabetes, obesity	36+1	(6/9) Preterm newborn	2925	450/none
**18–5502**	27	None	37+2	(7/9) Term newborn	2500	552/none
**16–3787**	24	None	37+4	(9/10) Term newborn	2945	461/none
**18–4510**	19	None	40+2	(7/8) Term newborn	1990	413/none
**18–6601**	16	Hypertensive disorder in pregnancy	24+5	(8/9) Preterm newborn	2235	368/none

**Table 3:** Clinical and morphological comparisons between COVID-19 group (n=19) and Control group (n=19) placentas.

		Variable		Control	COVID-19	*p*-value
**Clinical data**	**Maternal**	Maternal age (years)		25 (16–42)	32 (22–42)	0.21
		Gestational age (weeks)		36 (23–40)	36 (23–41)	0.97
		Comorbidities		Hypertensive disorder (3)Gestational diabetes (3) Obesity (3) Hypothyroidism (3) None (11)	Hypertensive disorder (3)Gestational diabetes (4) Obesity (4) Hypothyroidism (4) None (7)	1
	**Fetal**	APGAR 1 min/5 min		7 (1–9)/9 (6–10)	8(0–9)/9 (0–10)	0.41/0.33
		Fetal weight (grams)		2865 (1,180–3,810)	2663 (610–3,925)	0,94
		Placental Weight (grams)		406 (243–573)	412 (135–775)	0,79
		Placental diameter (centimeters)		17 (12–19)	16 (12–22)	0,87
		Infant death		0	3	**0.09**
		Preterm delivery		9	10	0.74
		Term delivery		10	9	0.74
**Morphological variables**	**MVM**	Villous infarction		5	2	0.21
		Distal villous hypoplasia		6	4	0.46
		Accelerated villous maturation/increase in syncytial knots		5	8	0.30
		Decidual vascular mural hypertrophy		5	10	**0.09**
		Absence of spiral artery remodeling		1	6	**0.03**
		Decidual vascular fibrinoid necrosis without foam cells		1	3	0.29
		Decidual vascular fibrinoid necrosis with foam cells		0	2	0.14
		Decidual arterial thrombosis		0	1	0.31
	**FVM**	Avascular villi small foci		1	1	1.0
		Avascular villi intermediate foci		1	0	0.31
		Fetal vascular thrombosis		1	6	**0.03**
		Fetal vascular thrombosis—umbilical cord		0	5	**0.02**
		Fetal vascular intramural fibrin deposition (non-occlusive)		0	2	0.14
		Vascular ectasia		2	1	0.54
	**DVM**	Delayed villous maturation (focal - <30%)		1	4	0.15
		Delayed villous maturation (extensive - >70%)		2	2	1.0
	**CI**	Chronic deciduitis—non-intense		17	16	0.31
		Chronic deciduitis—intense		1	0	0.29
		Villitis—low grade		1	1	1.0
		Chronic intervillositis—low grade		3	2	0.63
		Chronic intervillositis—high grade		1	2	0.54
	**MIR**	Subchorionitis/chorionitis		5	6	0.72
		Chorioamnionitis—non-intense		2	0	0.14
		Chorioamnionitis (necrosis)—intense		0	1	0.31
	**FIR**	Umbilical vasculitis		2	1	0.63
	**Others**	Intervillous thrombi		3	1	0.29
		Villous fibrin (focal—<30%)		15	14	0.70
		Villous fibrin (multifocal 30–70%)		3	5	0.42
		Villous fibrin (extensive—>70%)		1	0	0.31
		Villous edema		3	4	0.70
		Chorangiosis		4	3	0.42

p-value refers to the comparison between COVID-19 vs. the Control group; relevant values are highlighted (bold). p-value < 0.05. MVM, maternal vascular malperfusion; FVM, fetal vascular malperfusion; DVM, delayed vilous maturation; CI, chronic inflammation; MIR, maternal inflammatory response; FIR, fetal inflammatory response.

**Table 4:** Clinical and morphometrical comparisons between COVID-19 group (n=11) and Control group (n=11) placentas.

		Variable		Control	COVID-19	*p*-value
**Clinical data**	**Maternal**	Maternal age (years)	28.3 (18–42)		33 (26–42)	0.41
		Gestational age (weeks)	33.9 (23–39)		33.6 (23–38)	0.78
		Comorbidities	Hypertensive disorder (2) Gestational diabetes (2) Hypothyroidism (2) None (7)		Hypertensive disorder (2)Gestational diabetes (2)Hypothyroidism (2) None (4)	1
	**Fetal**	APGAR 1 min/5 min	6.5 (2–9)/8.7 (7–10)		6.6(0–9)/7 (0–10)	0.59/0.48
		Fetal weight (grams)	2656 (1,180–3,810)		2213 (610–3,030)	0.94
		Placental Weight (grams)	395 (243–573)		367 (135–650)	0.87
		Fetal/Placental ratio	6.74 (4.3–8.4)		5.96(3.63–7.55)	0.61
		Placental diameter (centimeters)	16.45 (13–19)		16.2 (12–20)	0.88
		Infant death	0		3	0.11
		Preterm newborn	7		6	0.66
		Term newborn	4		4	0.66
**Morphometric variables**	**HE**	Villi number	9.0 (5.3–14.5)		8.3 (4.7–10.9)	0.62
		Knots/villus	0.81 (0.6–1.0)		0.81 (0.6–1.0)	0.97
		Sprouts/villus	0.16 (0.1–0.3)		0.19 (0.1–0.5)	0.92
		Villus diameter	50.52 (43.4–58.8)		51.4 (45.5–63.0)	0.57
		Villus perimeter	288.42 (202.7–368.5)		271.93(213.8–358.4)	0.37
	**HPt**	Fibrin area (µm^2^)	485.36(61.9–1749.6)		686.64 (170.3–2053.0)	**0.08**
	**SiriusRed**	Collagen I percentage	41.01(10.1–66.1)		36.40(13.8–68.6)	0.45
		Collagen III percentage	58.99(33.9–89.9)		63.60 (31.4–86.2)	0.45
**Immunohistochemistry**		CD68+ Hofbauer cell/villous	1.2		1.7	0.12

p-value refers to the comparison between COVID-19 vs. the Control group; relevant values are highlighted (bold). p-value < 0.05. HPt, phosphotungstic hematoxylin.

### Maternal Clinical Profile and SARS-CoV-2 Testing Results

Among the nineteen patients testing positive for SARS-CoV-2 infection, almost half (9/19, 47.4%) were symptomatic. Three had COVID-19 symptoms varying from mild (fever, cough, among others, but without dyspnea) to moderate (with dyspnea, but without the necessity of complementary life support) (3/9, 33.3%). Six developed severe courses, requiring orotracheal intubation and hemodynamic support within less than seven days after admission (6/9, 66.6%). Two of them died due to COVID-19 associated complications (2/6, 33.3%).

Twelve patients of the COVID-19 group had at least one comorbidity (12/19, 63.2%), such as hypertension (3/19, 15.8%), diabetes (4/19, 21%), obesity (4/19, 21%), and hypothyroidism (4/19, 21%). One patient had a diagnosis of Kartagener syndrome (chronic sinusitis, bronchiectasis, and situs inversus with dextrocardia) and had a cardiac valvar replacement (metallic) for 10 years (Patient code—PC 20-3282).

Most pregnant women tested positive for SARS-CoV-2 in the third gestational trimester (16/19, 84.2%), either immediately before delivery in asymptomatic patients or within less than 15 days from delivery in symptomatic ones.

Only three patients had a positive result in the second trimester, all of them with severe COVID-19 symptoms and equally close to delivery. In this subgroup, one woman died due to COVID-19 complications along with her infant (PC 20-5379). One had an intrauterine demise (PC 20-3364) and recovered utterly afterward. The third evolved to preterm labor (PC 20-3561).

### Newborns Clinical Outcome and SARS-CoV-2 Testing Results

The mode of delivery, APGAR score, placental weight, fetal weight, fetal/placental weight ratio were similar between both groups (p=NS).

Among the 19 infants in the COVID-19 group, sixteen were born alive (16/19, 84.2%). We observed three infant deaths, being one intrauterine demise and two neonatal deaths within hours of delivery (3/19, 15.8%). All infant deaths occurred in women with severe COVID-19 symptoms, including the two who died due to COVID-19 complications (PC 20-3282 and 20-5379). In the Control group, all infants were born alive.

Preterm delivery was recorded in ten cases (10/19, 52.6%), including those with infant deaths mentioned above. In the Control group, nine pregnancies ended prematurely (9/19, 47.4%), a similarity that was expected due to methodological design. However, there were no recorded infant or maternal deaths.

SARS-CoV-2 RNA was detected in samples from five infants among thirteen tested (5/13, 38.4%). It was positive in one cord blood sample (PC 20-3364) and four newborns nasopharyngeal swabs specimens (PC 20-3282, 20-5379, 20-3744, and 20-5776). All amniotic fluid and mother’s milk samples tests returned negative.

From all FFPE placental tissue tested (n = 11), only one case was positive for SARS-CoV-2 RNA, being the case of intrauterine demise (PC 20-3364).

### Morphologic Alterations

All parameters enumerated in *Materials and Methods* were sought systematically. Many of them were not identified in any sample. The histopathological alterations identified in both groups are listed in **Table 3** and discussed below.

### Morphometric Alterations and Immunohistochemistry Evaluation

The average number of sprouts and knots (per villous) of the COVID-19 group tertiary villi was 0.19 and 0.81, respectively, compared to 0.16 and 0.81 of the Control group (*p*=NS). The average perimeter of the COVID-19 group tertiary villi was 271.93 µm compared to 288.42 µm in the Control group (*p*=0.37). The mean diameter of the COVID-19 group tertiary villi was 51.78 µm than 50.52 µm in the Control group (*p*=0.57).

The quantitative morphometric analysis of deposition of villous fibrin revealed an average area of 686.64 µm^2^ in the COVID-19 group compared to 485.36 µm^2^ in the Control group (*p*=0.08 - borderline).

The quantitative morphometric analysis of deposition of intra-villous collagen I and III revealed that COVID-19 group average areas were 36.40% and 63.60%, respectively, compared to 41.01% and 58.99% of the Control group (*p*=0.45).

HC counting revealed an average number of 1,7 CD68+ cell/villous in the COVID-19 group and 1,2 CD68+ cell/villous in the Control group (*p*=0.12).

## Discussion

New insights are being acquired on SARS-CoV-2 infection pathophysiology. COVID-19 is associated with an exaggerated inflammatory response, usually proportional to the disease’s severity, recognized as a cytokine storm (37). Such inflammatory alterations cause endothelial damage and disruption in the coagulation system, which may play a direct pathogenic role in the disease (38). Reports of hypercoagulability, with d-dimer elevation, development of ischemic changes as gangrene of extremities, and even disseminated intravascular coagulopathy, are not uncommon. There is emerging evidence that at least part of COVID-19 manifestations is associated with a systemic thrombotic and microvascular injury (39–45). In pregnant women, when coagulation is already altered by pregnancy itself, the impact of COVID-19 is still the object of study.

In this context, placental findings are invaluable. To date, histopathological alterations described encompass maternal vascular malperfusion (MVM) features, including low placental weight, accelerated villous maturation, decidual vasculopathy, and infarcts. The MVM findings are frequently observed in placentas from pregnant women with hypertensive disorders, such as gestational hypertension and preeclampsia, and have been associated with oligohydramnios, preterm birth, and stillbirth. Fetal vascular malperfusion (FVM) alterations have been described as well, such as focal thrombosis of fetal placental vessels (30–34, 46). However, various authors did not identify a correlation between placental lesions and maternal infection, notably when the samples analyzed were obtained from uninfected placentas and infants (47).

Interestingly, in cases with confirmed transplacental infection, inflammatory alterations were more frequently observed, particularly chronic histiocytic intervillositis with trophoblast necrosis. In these cases, SARS-CoV-2 was detected in the syncytiotrophoblast by immunohistochemistry and or RNA *in situ* hybridization (48, 49). It is not yet clear if the syncytiotrophoblast destruction is caused by a direct viral effect or is secondary to inflammatory or ischemic injury. Whichever mechanism is involved, the damage of this protective villous layer can facilitate fetal infection.

Recognition of the disease’s impact on the placenta, and the maternal-fetal response’s nature, may help understand the processes involved in pathogenesis, and ultimately, it may lead to an explanation for an adverse outcome.

### Maternal Clinical Profile and SARS-CoV-2 Testing Results

There were no significant differences in maternal age, gestational age at delivery, and maternal comorbidities profile between groups, thus corroborating that matching variables were adequately paired (p=NS).

In our COVID-19 group, the proportion of symptomatic patients is elevated (9/19, 47.4%). Since patients’ recruitment was done at the beginning of the pandemic, almost half of our cases correspond to symptomatic patients. After the universal screening was adopted, asymptomatic patients were also incorporated into the study.

In the literature, pregnant women’s outcomes have not been worse when compared to non-pregnant adult individuals. The severity of the COVID-19 seems to be related to existing comorbidities, like hypertension, obesity, among others (50–53), similarly to the general population. Most symptomatic patients in our study group had at least one comorbidity (8/9, 89%), including all the severely ill ones. In comparison, less than half of asymptomatic women had one comorbidity (4/10, 40%), and only one patient without comorbidities presented with mild symptoms (1/7, 15%—PC 20-5869).

Two women died from COVID-19 complications (PC 20-3282 and PC 20-5379). Both died shortly after hospitalization (three and one days after admission, respectively).

### Newborns Clinical Outcome and SARS-CoV-2 Testing Results

The newborn outcome was directly related to the mother’s health status among women with COVID-19. In patients with severe disease, the conceptus health deteriorated after Intensive Care Unit admission in all cases. Thus, not surprisingly, an adverse fetal outcome was more likely to occur in symptomatic patients (7/9, 77.7%) when compared to asymptomatic ones (3/10, 30%). Most of the asymptomatic patients had term deliveries (7/10, 70%) without recorded complications.

When considering patients with comorbidities (12/19, 63.2%), eight gestations ended prematurely (8/12, 66.6%). This proportion is slightly higher than what was observed in the Control group (5/9, 55.5%), even though clinical variables were adequately paired. Compared to the Control group, COVID-19 was also more frequently associated with maternal and infant deaths (although not statistically significant, p = 0.09).

Pertaining perinatal transmission of SARS-CoV-2, published reports to date suggest that it occurs, but is considered rare, with less than 2% of neonates with positive results until 24 h of life (54, 55).

Schwartz et al. proposed that positive samples for SARS-CoV-2 RNA collected within the initial 72 h of life can be considered diagnostic of early-onset COVID-19 infection. The probability that the infection resulted from the vertical transmission is even greater if the test is performed until 24 h of life (very early-onset COVID-19 infection). The authors also proposed that in cases where pregnant women and their neonates both tested positive for SARS-CoV-2, transplacental transmission could be confirmed by demonstrating the virus in fetal-derived placental tissue using immunohistochemistry to demonstrate SARS-CoV-2 antigens or RNA *in situ* hybridization to demonstrate viral nucleic acid (56). The World Health Organization recently published a manual with a definition and categorization of the timing of mother-to-child transmission of the SARS-CoV-2. Since various fetal samples are prone to cross-contamination, it preconizes rigorous methodology for sample gathering and strict criteria for establishing congenital transmission. Viral detection should be preferably performed in sterile samples, collected at birth, using nucleic acid detection techniques to confirm transplacental transmission (54).

From newborns’ nasopharyngeal swabs and umbilical cord blood specimens tested, five resulted positive and were considered a possible congenital infection, or very early-onset COVID-19 infection, by the criteria above.

In two cases, the newborns were healthy until hospital discharge (PC 20-3744 and 20-5776). Two cases resulted in short-term neonatal deaths (PC 20-3282 and 20-5379); the families did not authorize postmortem evaluation in both cases. None of them exhibited symptoms attributable to SARS-CoV-2 disease.

In the fifth positive infant, a stillborn, SARS-CoV-2 RNA was detected in the umbilical cord blood sample collected immediately after delivery and in the placental FFPE tissue (PC 20-3364). An autopsy was performed, and evaluation of fetal tissues showed mild microglial hyperplasia, mild lymphocytic infiltrate, and edema in skeletal muscle. Other findings were unspecific and probably caused by intrauterine asphyxia. All fetal tissue samples tested negative for viral RNA. The authors (57) previously reported those results.

### Morphologic Alterations

Our samples exhibited alterations spanning all major Amsterdam Placental Workshop Group Consensus Statement categories (MVM, FVM, delayed villous maturation, and inflammatory features). Most of them exhibited similar distribution between the two groups, which was expected due to the matching process. However, some results were unexpected.

Although maternal age and comorbidities were adequately matched, COVID-19 group placentas were more likely to show some MVM features when compared to controls, particularly signs of decidual vasculopathy. Decidual vascular mural hypertrophy was more frequently observed in the COVID-19 group (but did not reach statistical significance, p = 0.09). Ten patients exhibited this alteration (10/19, 56.6%), the majority without a recorded hypertensive disorder (7/10, 70%). In contrast, three of the five women with decidual vascular mural hypertrophy in the Control group had a hypertensive disorder.

The absence of spiral artery remodeling was significantly more frequent in the COVID-19 group (p=0,03). Six patients (6/19, 31.6%) exhibited this alteration combined with decidual vascular fibrinoid necrosis, mainly in women without the hypertensive disorder (5/6, 83.3%). In the Control group, those findings were noticed in only two cases, both in women with hypertension. Decidual arterial thrombosis was observed in only one case that ended with maternal and fetal death (PC 20-5379).

Accelerated villous maturation or increase in syncytial knots was similar in both groups, and those findings are supported by the morphometric analysis results discussed below. On the other hand, villous infarction and distal villous hypoplasia were less frequent in the study group.

Those findings are in consonance with previous reports describing higher decidual arteriopathy rates as a maternal vascular malperfusion feature in SARS-CoV-2 infected women. According to Shanes et al., though at least some of those alterations are thought to be chronic, its precise time of development is not precisely known, and as decidual arteriopathy appears to be more strongly related to COVID-19, it may be originating from a different mechanism (30).

Fetal vascular thrombosis was the FVM feature more frequently observed in our COVID-19 group. It was present in six cases (6/19, 31,5%), a significantly higher rate than in the Control group (p = 0.03). Of note, one case corresponded to the mother with Kartagener syndrome that evolved to maternal and neonatal death (PC 20-3282). In the Control group, this finding was detected only in one case, the mother showing no comorbidity (PC 20-5502). The distal lesions in villi indicative of fetal malperfusion were similar between both groups.

The frequency of inflammatory changes was similar between groups. Chronic histiocytic intervillositis, characterized by the accumulation of histocytes in the intervillous space, belongs to this category. In the context of COVID-19, such alteration is not frequently reported, and when present, was associated with adverse fetal outcomes and or with documented newborn infection by SARS-CoV-2 (30–33, 48, 49).

We identified chronic histiocytic intervillositis in four of our cases (PC 20-3282, PC 20-5379, PC 20-3364, and PC 20-4850). All four mothers with placentas having this finding had a severe COVID-19 course; two of them died. Their infants were prematurely born; three of them were positive for SARS-CoV-2 RNA in nasopharyngeal swab or umbilical cord blood samples. Those three died as well.

In the three cases that resulted in maternal and or infant death (PC 20-3282, PC 20-5379, PC 20-3364), features of MVM, FVM, and inflammatory changes were identified in various combinations. However, only in one case (PC 20-3364), MVM and FVM features were more intense and exhibited a broader distribution throughout the placenta than other specimens from both groups. This patient had a hypertensive disorder as well; because of that, those alterations cannot be attributed entirely to viral injury, even though COVID-19 may have a contributory role in the pathophysiology. Of note, chronic histiocytic intervillositis was observed in all three, two of them categorized as high grade (PC 20-5379, PC 20-3364).

Considering only the two cases that resulted in maternal deaths, placental findings were similar to those observed in the COVID-19 group, except for the presence of chronic histiocytic intervillositis.

Although the frequency of chronic histiocytic intervillositis was similar in COVID-19 and Control groups, this finding was not associated with adverse outcomes in the Control group. Interestingly, in the COVID-19 group, this finding was more frequently observed in placentas of severely ill patients, including those that died from COVID-19 complications, those associated with infant deaths, and with a positive SARS-CoV-2 RNA test in fetal tissues.

### Morphometric Alterations and Immunohistochemistry Evaluation

Measurements of villi, such as diameter and perimeter, and counting of sprouts and knots in tertiary villi, aimed to evaluate villi maturity objectively (58, 59). Changes found in the placentas of the COVID-19 group do not seem to be different from those observed in the Control group, as far as maternal age, gestational age, and comorbidities are paired. Those data corroborate the morphological impression that villous maturity retardation or acceleration was not different between our COVID-19 and Control groups.

Fibrosis can be the final event after villi damage, following inflammatory, infectious, or vascular insults, such as described in FVM physiopathology (60). The relative amount of villous fibrosis estimated by evaluation of collagen I and III depositions with Sirius Red histochemical stain showed no difference between groups. There was no difference between groups for global collagen deposition analysis as well, meaning that there was no relative increase in the amount of villous fibrosis in the COVID-19 group.

The amount of fibrin deposited in the villi evaluated by the phosphotungstic hematoxylin histochemical stain could be more accentuated in the COVID-19 group since the difference between groups was borderline. However, such borderline difference between COVID-19 and Control groups was not perceived at the morphological analysis. In the qualitative evaluation, fibrin deposition seemed to be similarly increased in both groups, both in perivillous and intravillous topography.

The number of HC per tertiary villi was also similar between groups. This finding suggests that, at least in perinatally infected women, villous histocytes did not proliferate, and HC hyperplasia may not be as involved in the physiopathology of COVID-19 in the placenta as described for other viruses like Zika virus or HIV (28, 61–63).

In conclusion, pregnant women with symptomatic SARS-CoV-2 infection, particularly with the severe course, are more likely to exhibit an adverse fetal outcome, with slightly more frequent histopathologic findings of maternal and fetal vascular malperfusion, and chronic histiocytic intervillositis. The morphometric changes found in the placentas of the COVID-19 group do not seem to be different from those observed in the Control group, as far as maternal age, gestational age, and comorbidities are paired. Only the deposition of villous fibrin could be more accentuated in the COVID-19 group (p = 0.08 borderline). The number of HC/villous evaluated with CD68 immunohistochemistry did not show a difference between both groups.

## Data Availability Statement

The original contributions presented in the study are included in the article/supplementary material. Further inquiries can be directed to the corresponding authors.

## Ethics Statement

The studies involving human participants were reviewed and approved by Comitê de Ética em Pesquisa em Seres Humanos do Hospital de Clínicas da Universidade Federal do Paraná. The patients/participants provided their written informed consent to participate in this study.

## Author Contributions

DP, AO, and VD contributed to collecting SARS-CoV-2 patients’ samples and medical records in HNSG. IF and JL contributed to the collection of SARS-CoV-2 patients’ samples and medical records in CHC-UFPR. AZ and AF contributed to the RNA extraction and RT-PCR reactions in HNSG. MN, BC, and CD were responsible for the RT-PCR reactions in CHC-UFPR. ES was responsible for selecting the controls and contributed to the immunohistochemistry analysis. PR was responsible for morphological and morphometrical analysis, interpretation of data, and drafted the manuscript. JD and EH contributed to the literature review and morphometrical analysis (Phosphotungstic hematoxylin). FD contributed with morphometrical analysis (Sirius Red). AP contributed with morphological analysis. SR supported the experiments and contributed to the RT-PCR reactions in CHC-UFPR. CM-S supported the experiments and was responsible for the statistical analysis. NC contributed to the manuscript revision. LN supported the experiments, supervised the project, and was a significant contributor to the manuscript review. All authors contributed to the article and approved the submitted version.

## Funding

This research was funded by productivity research level 2 of the National Council for Scientific and Technological Development (CNPq) and by Pontifícia Universidade Católica do Paraná with resources from BRDE, Banco Regional de Desenvolvimento do Extremo Sul.

## Conflict of Interest

The authors declare that the research was conducted in the absence of any commercial or financial relationships that could be construed as a potential conflict of interest.
